# Exosomes in the Field of Neuroscience: A Scientometric Study and Visualization Analysis

**DOI:** 10.3389/fneur.2022.871491

**Published:** 2022-05-17

**Authors:** Junzi Long, Yasu Zhang, Xiaomin Liu, Mengyang Pan, Qian Gao

**Affiliations:** School of Rehabilitation Medicine, Henan University of Chinese Medicine, Zhengzhou, China

**Keywords:** exosomes, field of neuroscience, Alzheimer's disease, Parkinson's disease, multiple sclerosis, scientometrics, key words analysis, co-citation analysis

## Abstract

Exosomes have received great attention for their diagnostic, therapeutic, and prognostic roles in the field of neuroscience over the past decade. This scientometric study aimed to quantitatively and qualitatively evaluate knowledge structure, hot topics, and research trends of studies about exosomes in the field of neuroscience using visualization tools. Web of Science Core collection databases were searched for relevant publications between 2005 and 2021. The Carrot2 online system, BICOMB, gCLUTO, and Ucinet software were utilized for key word analysis, and co-citations analyses were conducted in Citespace and VOSviewer. Altogether, 21 high-frequency key words were collected from 856 included articles, and 5 clusters were identified through biclustering analyses. The strategic diagram and social network analysis further determined research hotspots and trends. Co-citation analysis results revealed a few crucial works that contributed to the development of research on exosomes in the field of neuroscience. Moreover, the important sources that had contributed to the development of this field were identified. Our findings suggested that Alzheimer's disease-related research remained a hot topic in this field till now, and recent researchers had extended their scopes to more cognitive impairments. Importantly, researches related to exosomes in multiple sclerosis and Parkinson's disease were promising. While exosomes in acute central nervous system injury had not been sufficiently investigated, with continuous improvement in exosome-based delivery technology, this subject might make a breakthrough in terms of therapeutic innovations in the immediate future.

## Introduction

Extracellular vesicles are membranous structures secreted by a majority of cells, comprising exosomes and microvesicles (1). As the smallest extracellular vesicles (50–150 nm in diameter), exosomes have recently gained intense interest for their roles in intercellular communications (2). Intracellularly, exosomes originate from the budding of the plasma membrane that ultimately matures into multivesicular bodies. Upon fusion with the cytomembrane, multivesicular bodies release exosomes into the microenvironment, carrying particular proteins, lipids, and genetic materials (3). Exosomes are currently considered to remove waste materials from cells to maintain cellular homeostasis, but the precise physiological significance remains unknown (4).

Research about exosomes in the field of neuroscience opened up in recent years. It has been established that in the central nervous system, both nerve and glial cells take the advantages of secreted exosomes to mediate interneuronal communication (5). Exosomes-mediated molecule exchange and signal transduction partly participate in the formation of new neural circuits and promotion of synaptic plasticity, as well as learning and memorizing procedures (6). In pathological conditions, however, the same molecular mechanism can facilitate the invasion and migration of nerve tumor cells and the spread of neurotoxic aggregates (7, 8). This prominent transmission capacity has also suggested that exosomes can be exploited in some clinical applications, including biomarkers and therapeutic carriers (9, 10). Overall, exosome-related researches have promoted the development of neuroscience based on their diagnostic, therapeutic, and prognostic potentials.

Scientometrics, also known as “the science of science, combines mathematical, bibliographical, and statistical methods for quantitative analyses of all knowledge carriers, which has advantages of evaluating academic achievements and predicting research trends (11). Many visualization tools have been developed for scientometric studies over recent years, which enable the improvement of timeliness, accessibility, and reproducibility of documentary research (12). In this review, we firstly employed scientometric methodologies and comprehensive visualization tools to analyze literature related to exosomes in the field of neuroscience, aiming to illuminate current situations and development trends in this discipline.

## Materials and Methods

### Data Collection

Relevant literature were retrieved from the Web of Science Core Collection using the following search strategy: TS = (exosome^*^). Then retrieved results were refined by the category of “neuroscience” on Web of Science. This search strategy was limited to published English papers between January 2005 and December 2021. The final results were saved as a plain text file with full records and cited references.

### Key Word Analysis

To identify key topics about exosomes in the field of neuroscience, the Carrot2 online system (https://search.carrot2.org/), a clustering and visualization platform, was utilized to organize retrieved articles into some topics based on their titles (13). The Bibliographic Item Co-Occurrence Matrix Builder (BICOMB) software (version 2.01, China Medical University) (14) was utilized to categorize retrieved bibliographic information and create their matrix relations via statistical algorithms. High-frequency words were identified to construct the binary matrix and co-word matrix. Binary matrix results were visualized using Graphical Clustering Toolkit (gCLUTO) 1.0 version software, which was a cross-platform graphical application for analyzing characteristics of various clusters, developed by professor Rasmussen and Karypis from Minnesota University (15). As for clustering options, we chose the repeated bisection, cosine for similarity function, and I2 for criterion function. An optimal clustering number was obtained by comparing the external and internal similarities of different clusters. Based on double clustering analysis results, mountain and matrix visualizations were conducted to summarize the hotspots of exosomes in neuroscience. Moreover, a strategic diagram was designed in Microsoft Excel through the calculations of co-word matrix results. Clusters in the strategic diagram were in accordance with the clustering results from gCLUTO. Furthermore, the Ucinet 6 software and its plug-in Netdraw (16) were used to perform a social network analysis through the whole network and individual node metrics, as well as visualization of data networks. Information of the overall network and individual modules were collected, such as density and degree.

### Co-citation Analysis

CiteSpace software combined techniques of scientometric analysis, visualization approaches, and data mining algorithms, and this study utilized CiteSpace 5.8 (Chaomei Chen, Drexel University) to analyze the co-citation network of included articles (17). The top 50 most cited articles in each year were selected to create a co-citation network without line pruning. Co-citation analysis grouped co-cited articles according to their similarities, and identified the main topics of each cluster. Frequent citations always indicated a common topic between co-cited articles. The relationship between citing articles and co-cited articles is illustrated in Supplementary Figure 1. A timeline view from 2005 to 2021 and references with strong citation bursts were also drawn by the CiteSpace 5.8. Moreover, VOSviewer (Leiden University, Netherlands) software (18), a visualization tool for scientometric analysis and knowledge domain mapping, was used to create a journal density visualization according to the analyses of citation and co-citation.

## Results

**Figure 1:**
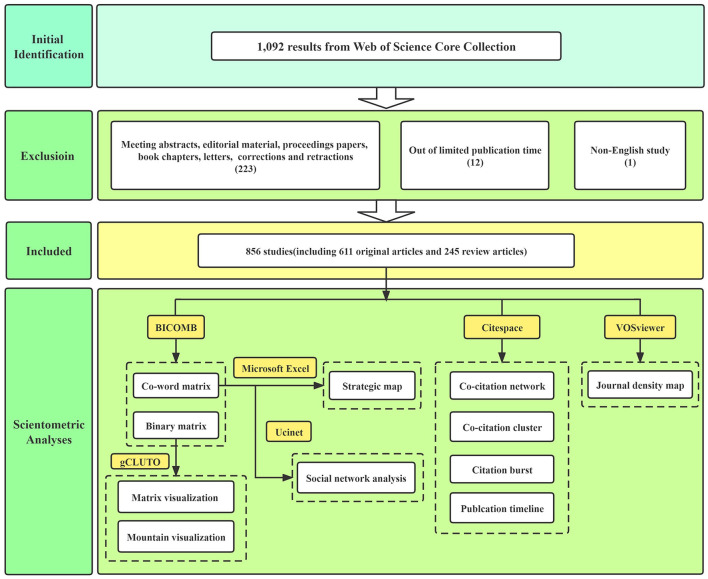
Flow chart of screening processes and scientometric analyses.

**Figure 2:**
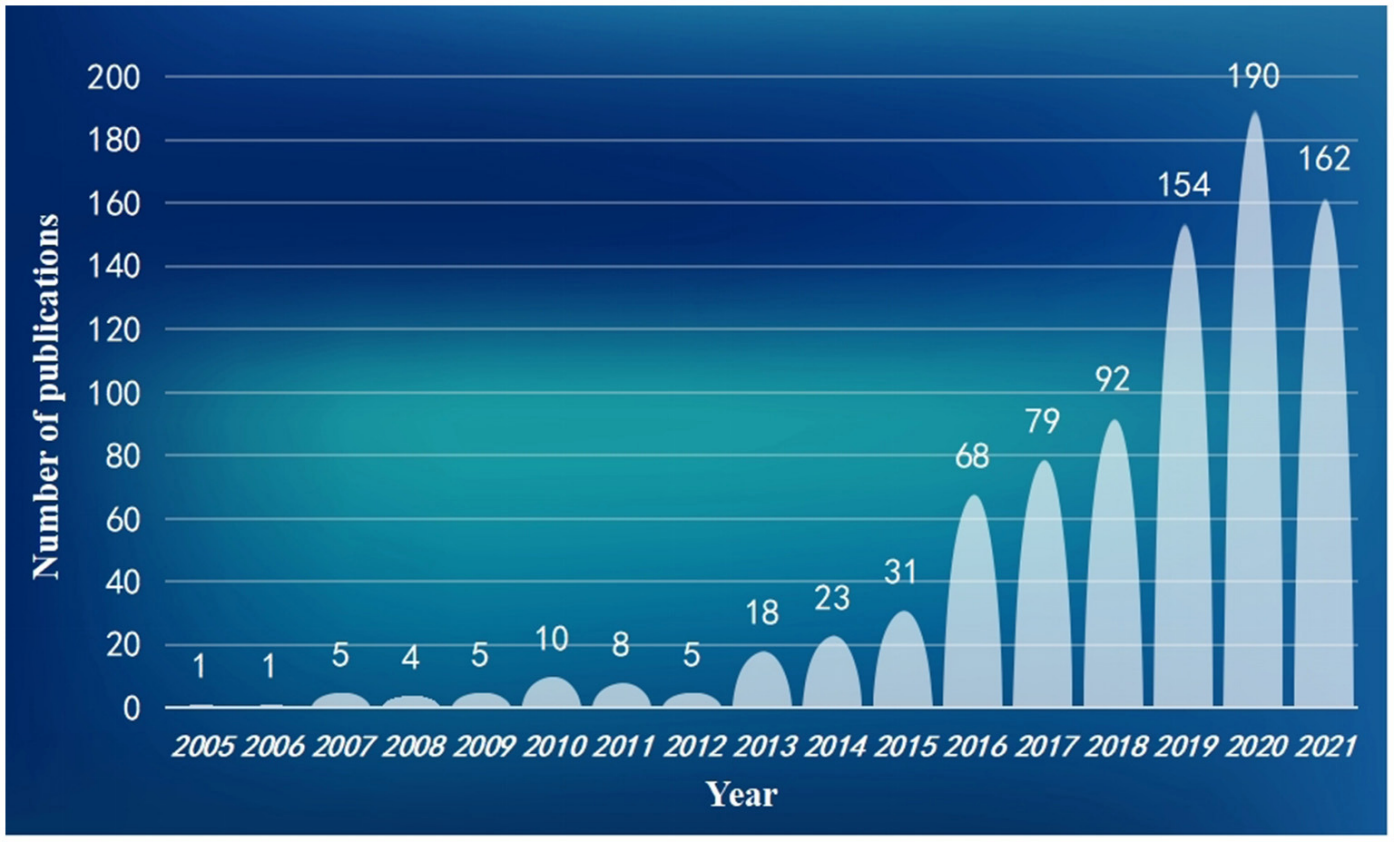
Publication trends on exosomes in the field of neuroscience from 2005 to 2021.

The electronic search yielded a total of 1,092 articles through Web of Science Core Collection databases. After literature identification and screening, a total of 856 studies matched the criteria for further analyses. Article screening processes and detailed scientometric analyses are displayed in Figure 1. Further, the yearly number of relevant publications is shown in Figure 2, suggesting a substantial increasing attention to exosomes in the field of neuroscience over time.

### Key Word Analysis Results

**Figure 3:**
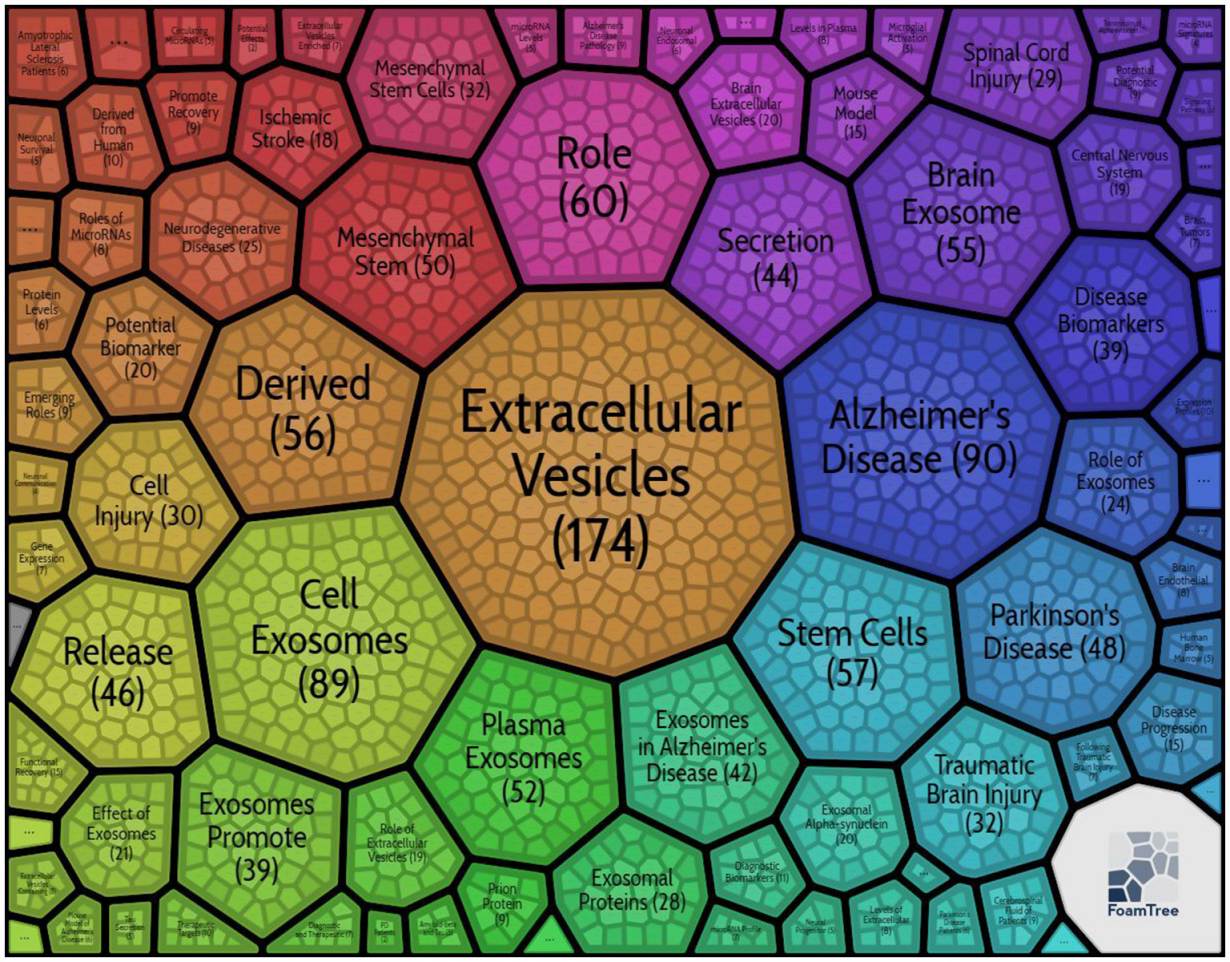
Eight-six key words about exosomes in the field of neuroscience-based on titles clustering results.

Based on the Carrot2 system, 86 key words were summarized from 856 titles (Figure 3), and the top 5 key words were extracellular vesicles (174), Alzheimer's disease (90), cell exosomes (89), role (60), and stem cells (57). In terms of overall key word analysis, we collected 2,179 kinds of words, amounting to 4,522, using Bicomb software after integrating synonyms, singular and plural forms, and uppercase and lowercase letters. The top 21 key words were considered as high-frequency words, because their frequency numbers were greater than their ranks (Table 1). Exosomes, extracellular vesicles, biomarkers, Alzheimer's disease, and MicroRNA were top five high-frequency words. Based on the 21 high-frequency words, we established a co-word matrix that showed co-occurrence frequency between these words (Supplementary Table 1). Supplementary Table 2 displayed a binary matrix of high-frequency terms, with columns representing source articles. The “0” meant words were not in the article and the “1” meant words were present.

**Table 1:** High-frequency words from publications on exosomes in the field of neuroscience (frequency ≥ 20).

**Rank**	**Key words**	**Frequency**	**Proportion(%)**
1	Exosomes	490	10.8359
2	Extracellular vesicles	186	4.1132
3	Biomarkers	113	2.4989
4	Alzheimer′s disease	111	2.4547
4	MicroRNA	111	2.4547
5	Parkinson′s disease	64	1.4153
6	Neurodegeneration	61	1.3490
7	Microglia	57	1.2605
8	Alpha-synuclein	44	0.9730
9	Neuroinflammation	37	0.8182
10	Inflammation	33	0.7298
11	Traumatic brain injury	31	0.6855
11	Spinal cord injury	31	0.6855
12	Microvesicles	30	0.6634
12	Astrocytes	30	0.6634
12	Tau	30	0.6634
13	Stroke	27	0.5971
14	Multiple sclerosis	21	0.4644
15	Mesenchymal stem cells	20	0.4423
15	Blood-brain barrier	20	0.4423
15	Central nervous system	20	0.4423

**Figure 4:**
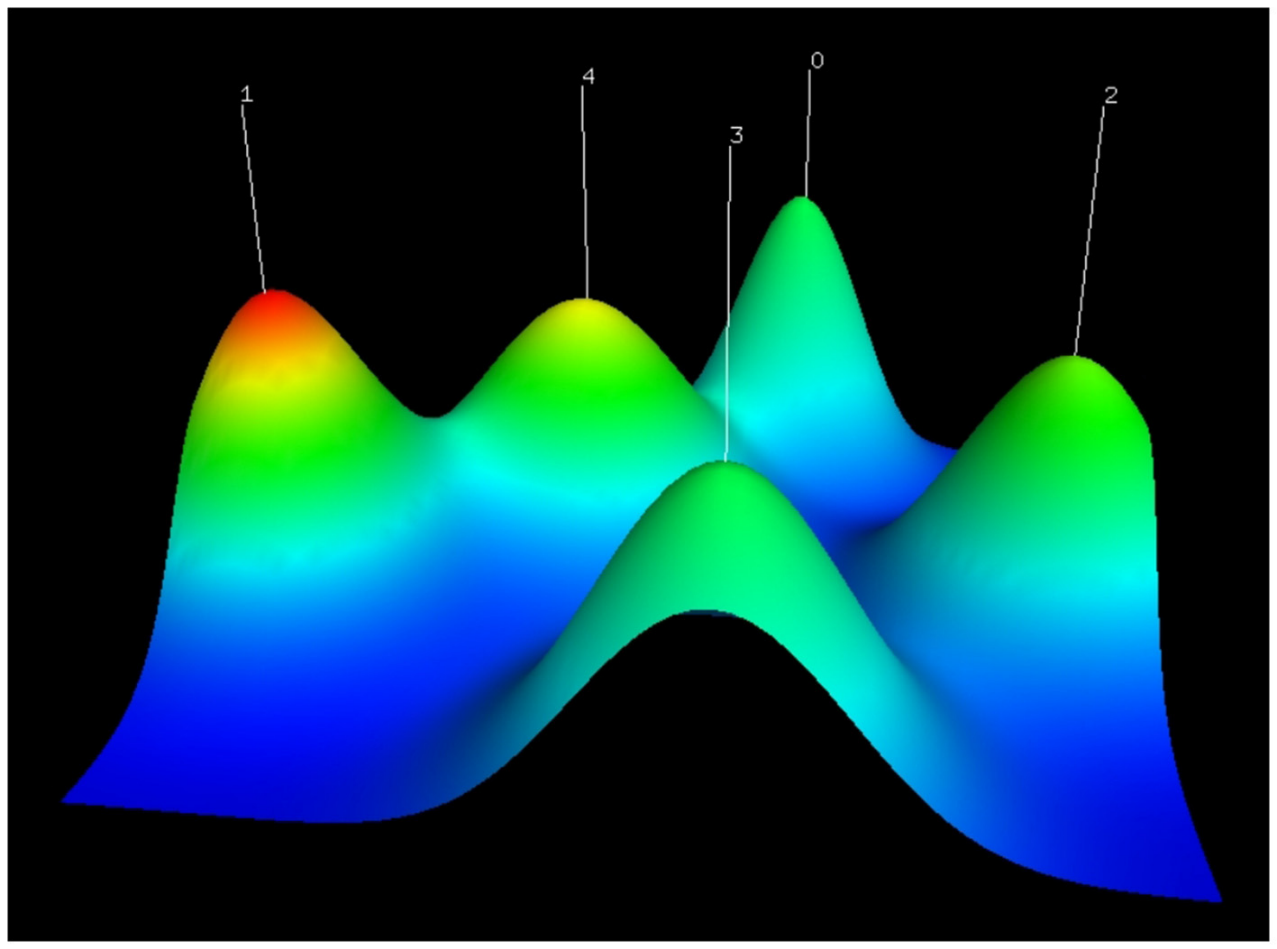
Mountain visualization of biclustering analysis results of high-frequency words about exosomes in the field of neuroscience.

As shown in Figure 4, double clustering analysis results for binary matrix were visualized by a mountain map. The color of the peak was proportional to standard deviation within cluster—warm color represented low standard deviation and cold color represented high standard deviation. As a result, clusters 1 and 4 had relatively low standard deviation, and clusters 0, 2, and 3 had relatively high standard deviation. A greater volume of a mountain contained more key words, and their altitudes were in direct proportion to internal similarity. Relatively high altitudes of five clusters demonstrated that they all had good internal similarities. The distance between two peaks represented their relative similarities, which meant that clusters 0, 1, and 4 were closely related to each other, while clusters 2 and 3 were relatively independent fields.

**Figure 5:**
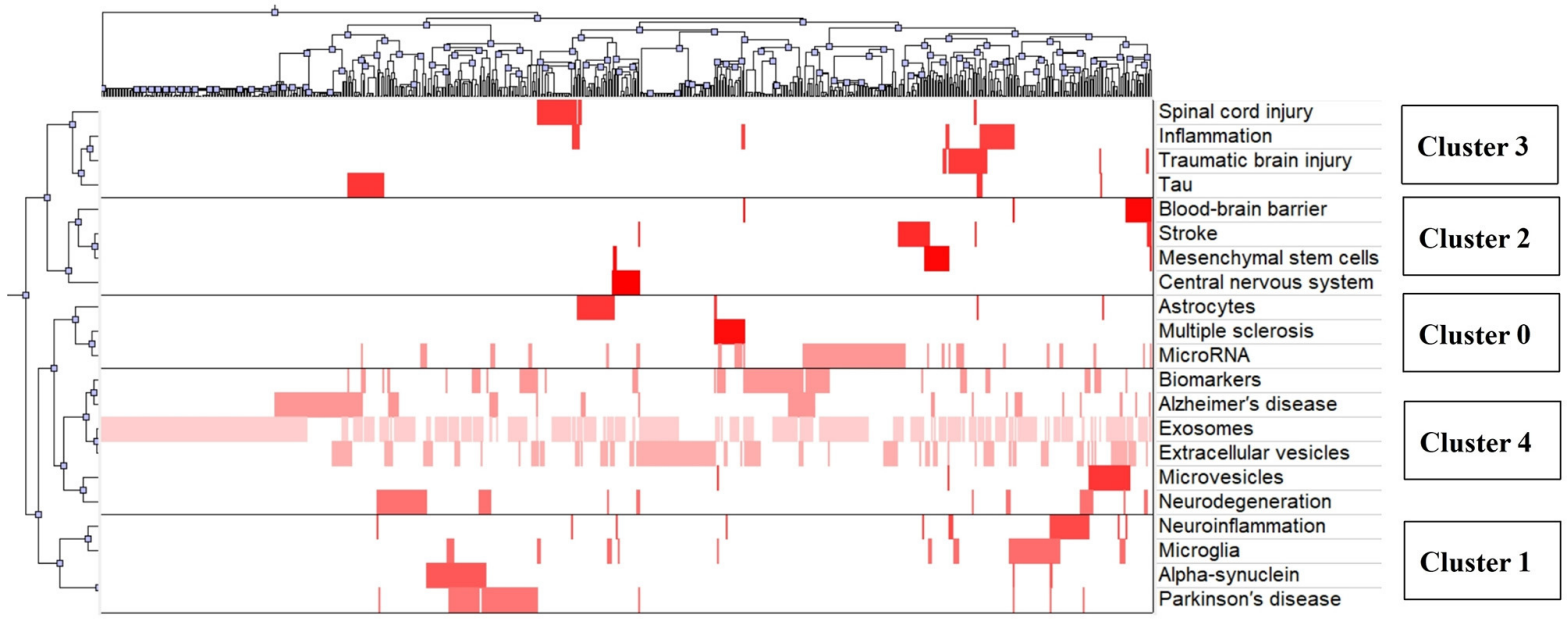
Matrix visualization of biclustering analysis results of high-frequency words about exosomes in the field of neuroscience.

In the visualized matrix (Figure 5), five clusters were listed on the right side, and each row represented a high-frequency word. Each above column represented an article. If a high-frequency word was included in the corresponding article, intersecting areas would be shown in red, where the higher the occurrence, the deeper the color. Cluster 4 consisted of many top high-frequency words, so it had the strongest correlation with our topics. The double clustering analysis parameters of five clusters are shown in Table 2. According to the disease in every cluster, we defined these clusters as follows:

**Table 2:** 5-way clustering results.

**Cluster**	**Size**	**ISim^**a**^**	**ISdev^**b**^**	**ESim^**c**^**	**ESdev^**d**^**
0	3	0.396	0.022	0.056	0.030
1	4	0.374	0.052	0.053	0.015
2	4	0.318	0.031	0.042	0.009
3	4	0.318	0.023	0.047	0.016
4	6	0.319	0.039	0.085	0.049

a *ISim, internal similarity;*

b *ISdev, internal similarity deviation;*

c *ESim, external similarity;*

d *ESdev, external similarity deviation*.

Cluster 0: Exosomes in the field of multiple sclerosis.

Cluster 1: Exosomes in the field of Parkinson's disease.

Cluster 2: Exosomes in the field of stroke.

Cluster 3: Exosomes in the field of spinal cord injury and traumatic brain injury.

Cluster 4: Exosomes in the field of Alzheimer's disease.

**Figure 6:**
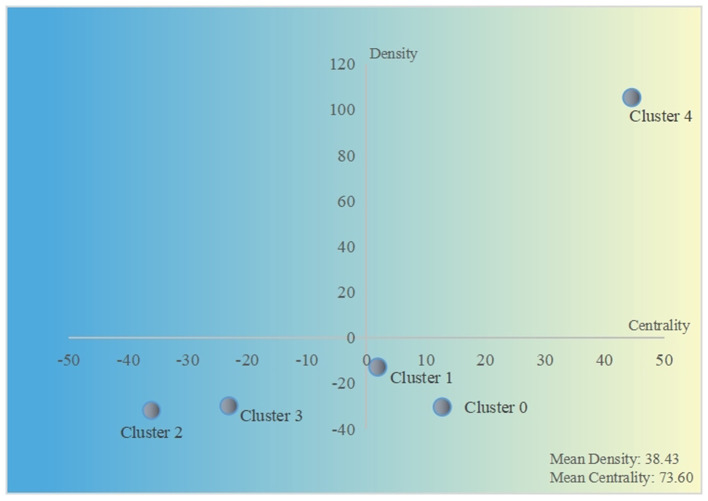
Strategic diagram of 5 clusters.

Based on the co-word matrix, a strategic diagram of 5 clusters was constructed (Figure 6). The horizontal axis represented cluster centrality, and the vertical axis represented cluster density. Cluster 4 had high centrality and density in the first quadrant, indicating that topics of exosomes in the field of Alzheimer's disease were very mature and hot. The second quadrant always represented a well-developed field but received low attention. Clusters 2 and 3 were in the third quadrant with low density and centrality, suggesting that their corresponding topics were unpopular or emerging. In the fourth quadrant, topics represented by clusters 0 and 1 might be promising and developing, which often interested researchers.

**Figure 7:**
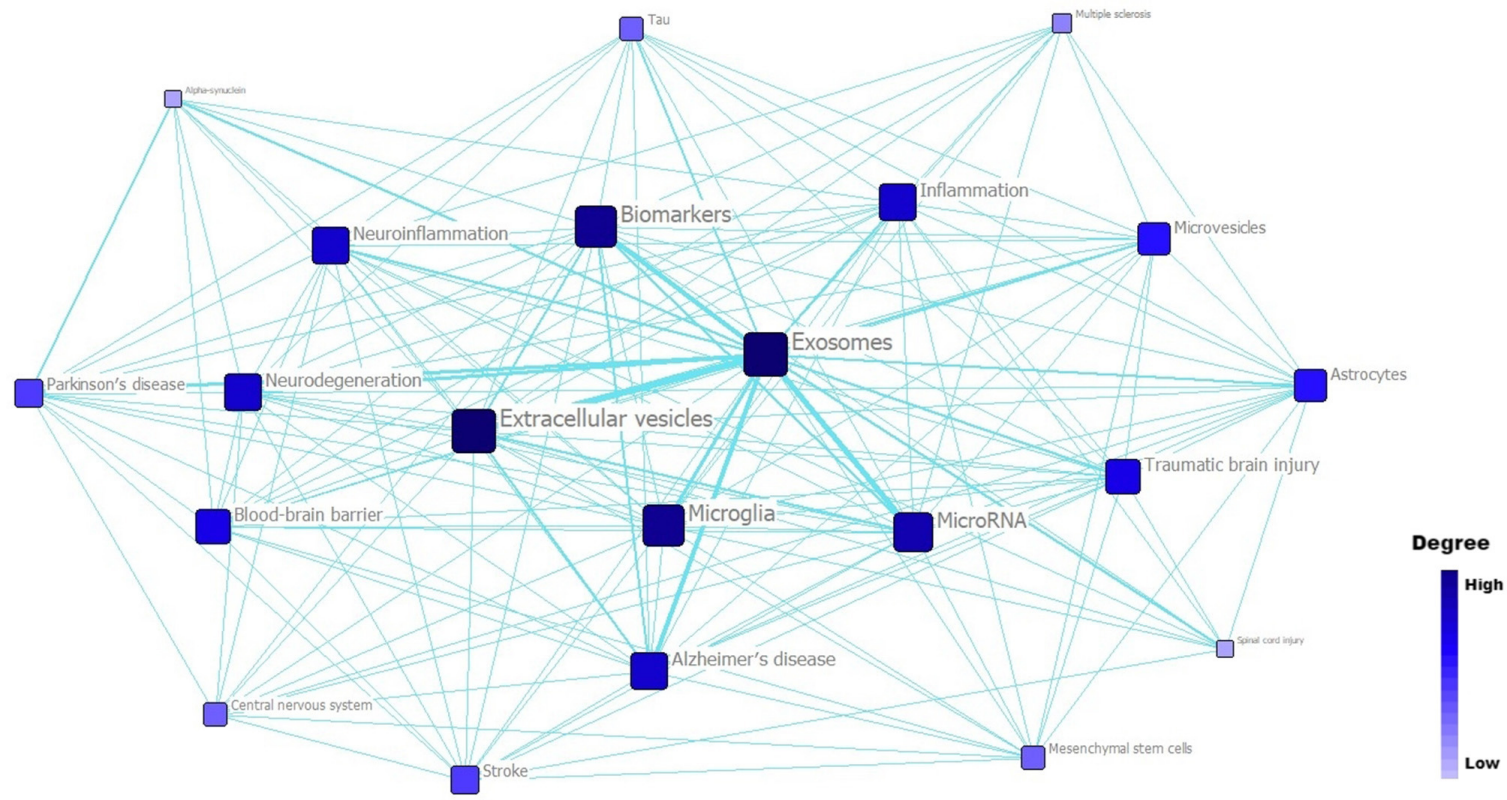
Social network analysis of top 21 high-frequency words related to exosomes in the field of neuroscience.

Furthermore, we performed a social network analysis of these high-frequency words using Ucinet software (Figure 7, Table 3). This network had an average degree of 15.143, a density of 0.757, an average distance of 1.243, and a SD distance of 0.429. As shown in Figure 7, the color depth and module size were proportional to a degree, and a thicker line reflected a closer relation between two modules (e.g., exosomes and microRNA, exosomes and microglia, and exosomes and Alzheimer's disease). Degree, closeness centrality, and betweenness centrality were considered for the analysis of high-frequency word network (Table 3). In short, closeness centrality measured the shortest distance between two modules, and betweenness centrality showed the intermediary role of each module. In this network, the words “exosomes,” “extracellular vesicles,” “microglia,” “biomarkers,” and “neuroinflammation” had a relatively high degree and betweenness centrality and low closeness centrality, so they were of vital importance to relevant researches.

**Table 3:** Social network parameters of 21 high-frequency words (ranked by degree).

**High-frequency words**	**Degree**	**Closeness centrality**	**Betweenness centrality**
Exosomes	20	20	5.8648386
Extracellular vesicles	20	20	5.8648386
Biomarkers	19	21	4.894203663
Microglia	19	21	4.932658672
MicroRNA	18	22	3.008091688
Alzheimer′s disease	17	23	2.047377586
Neurodegeneration	17	23	2.594203234
Neuroinflammation	17	23	4.126370907
Inflammation	17	23	4.055627823
Traumatic brain injury	16	24	2.038142204
Blood-brain barrier	16	24	2.425865889
Microvesicles	15	25	1.342832208
Astrocytes	15	25	2.499639273
Parkinson′s disease	14	26	1.370310307
Stroke	14	26	1.529834032
Tau	12	28	0.417832166
Mesenchymal stem cells	12	28	0.730302989
Central nervous system	12	28	0.645609915
Multiple sclerosis	10	30	0.226923078
Alpha-synuclein	9	31	0.06666667
Spinal cord injury	9	31	0.317832172

### Co-citation Analysis Results

**Figure 8:**
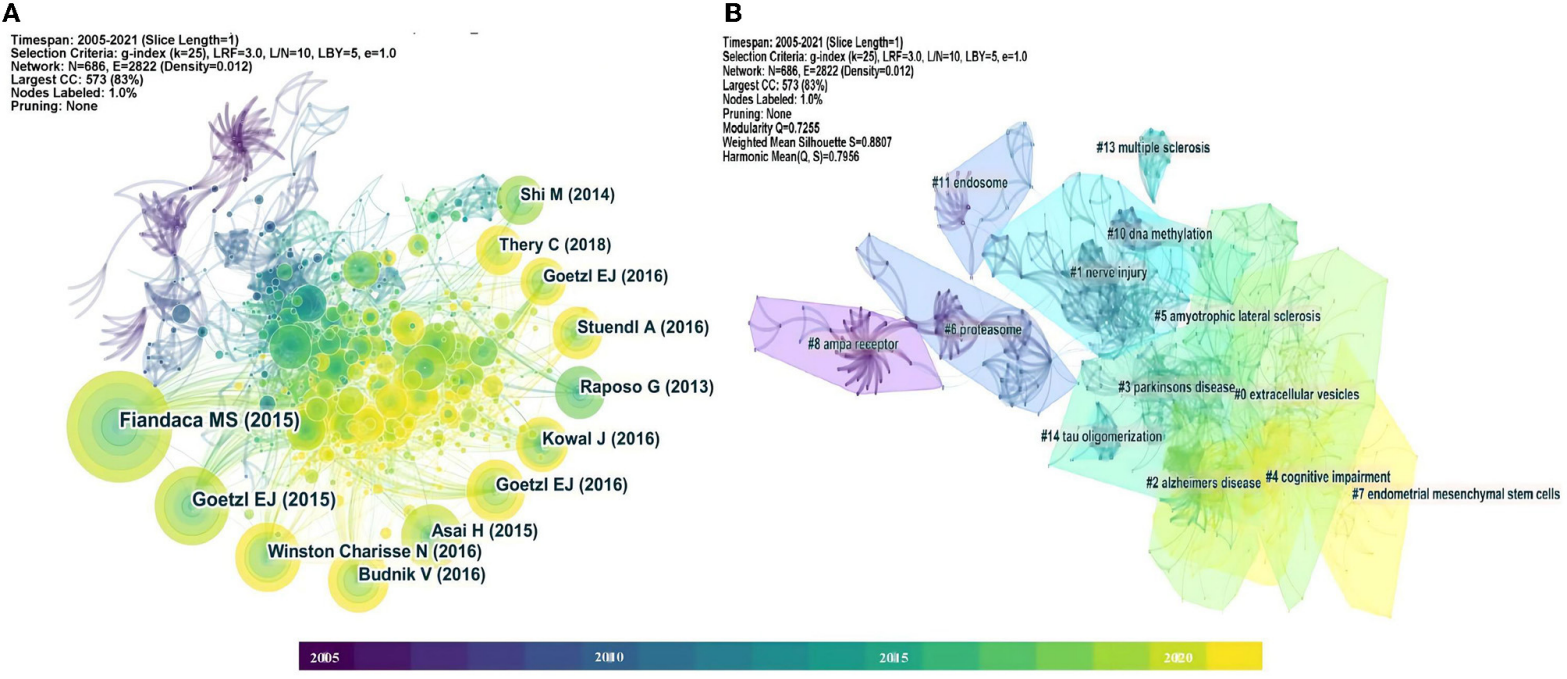
**(A)** Co-citation analysis network about exosomes in the field of neuroscience. **(B)** Clustering analysis of co-citation network.

Co-citation analysis is of great significance for researchers to understand the development trend and basic theory of a certain field. As displayed in Figure 8A, the co-citation network map contained 686 nodes and 2,822 lines. Node radius was proportional to its cited frequency, and each ring represented 1 year. The links connected to co-cited articles. From this co-citation analysis results, some great contributions about exosomes in the field of neuroscience have been revealed. The top 12 most-cited articles (frequency ≥ 24) are summarized in Table 4. The study by Fiandaca et al. titled “Identification of preclinical Alzheimer's disease by a profile of pathogenic proteins in neurally derived blood exosomes: a case-control study” (19) had the highest frequency among included articles, which could be considered as a crucial work about exosomes in Alzheimer's disease. Besides, four other articles on the role of exosomes in Alzheimer's disease also had high frequency (20, 21, 24, 28). Two experimental studies focused on cerebrospinal fluid exosomes from Parkinson's disease patients (27, 29). Moreover, four researchers investigated the characteristics and functions of exosomes (22, 23, 25, 26). An updated minimal information for studies of extracellular vesicles guideline was also included, proposed by the international society for extracellular vesicles (30).

**Table 4:** Summary of the top 12 most-cited articles about exosomes in the field of neuroscience from the co-citation analysis results.

**Author (year)**	**Frequency**	**Title**	**Journal**	**Main ideas**
Fiandaca et al. (19)	54	Identification of preclinical Alzheimer's disease by a profile of pathogenic proteins in neurally derived blood exosomes: a case-control study	Alzheimers & Dementia	Some pathogenic proteins in neurally derived blood exosomes can predict the development of Alzheimer's disease
Goetzl et al. (20)	38	Altered lysosomal proteins in neural-derived plasma exosomes in preclinical Alzheimer's disease	Neurology	Autolysosomal proteins in neurally derived blood exosomes can distinguish Alzheimer's disease patients from case controls
Winston et al. (21)	33	Prediction of conversion from mild cognitive impairment to dementia with neuronally derived blood exosome protein profile	Alzheimers & Dementia	Abnormal levels of some proteins in plasma neuronal derived exosomes accurately predict conversion of mild cognitive impairment to Alzheimer's disease
Budnik et al. (22)	32	Extracellular vesicles round off communication in the nervous system	Nature Reviews Neuroscience	Extracellular vesicles work as intercellular communication devices within the nervous system
Asai et al. (23)	31	Depletion of microglia and inhibition of exosome synthesis halt tau propagation	Nature Neuroscience	Microglia can spread tau via exosome secretion
Goetzl et al. (24)	30	Decreased synaptic proteins in neuronal exosomes of frontotemporal dementia and Alzheimer's disease	FASEB Journal	Neuronal-derived exosomes synaptic proteins may be useful preclinical indices and progression measures in senile dementia.
Kowal et al. (25)	27	Proteomic comparison defines novel markers to characterize heterogeneous populations of extracellular vesicle subtypes	Proceedings of the National Academy of Sciences of the United States of America	Extracellular vesicles include exosomal and nonexosomal subpopulations, and their differential separations can be reached by immuno-isolation way
Raposo et al. (26)	26	Extracellular vesicles: exosomes, microvesicles, and friends	Journal of Cell Biology	This study reviews the characterization, formation, targeting, and function of extracellular vesicles
Stuendl et al. (27)	26	Induction of α-synuclein aggregate formation by CSF exosomes from patients with Parkinson's disease and dementia with Lewy bodies	Brain	Cerebrospinal fluid exosomes from patients with Parkinson's disease and dementia with Lewy bodies contain a pathogenic species of α-synuclein
Goetzl et al. (28)	24	Cargo proteins of plasma astrocyte-derived exosomes in Alzheimer's disease	FASEB Journal	Astrocyte-derived exosomes cargo proteins may be useful to investigate cellular interactions and effects of β-site amyloid precursor protein-cleaving enzyme 1 inhibitors in Alzheimer's disease
Shi et al. (29)	24	Plasma exosomal α-synuclein is likely CNS-derived and increased in Parkinson's disease	Acta Neuropathological	Cerebrospinal fluid α-synuclein contained in exosomes can be readily transported to blood, especially in Parkinson's disease patients
Thery C (30)	24	Minimal information for studies of extracellular vesicles 2018 (MISEV2018): a position statement of the International Society for Extracellular Vesicles and update of the MISEV2014 guidelines	Journal of Extracellular Vesicles	This guideline updates the International Society for Extracellular Vesicles (ISEV) proposed Minimal Information for Studies of Extracellular Vesicles guidelines for the field in 2014

Figure 8B showed 13 cluster visualizations of the co-citation network based on key words, and clusters 9 and 12 were removed because of their long distance from the center (unimportant). Cluster labels were defined using latent semantic indexing (LSI). The modularity *Q* was 0.7255 and the weighted mean silhouette was 0.8807, which suggested convincing clustering results. The top five clusters included “extracellular vesicles,” “nerve injury,” “Alzheimer's disease,” “Parkinson's disease,” and “cognitive impairment.” “AMPA receptor” was an earlier cluster, while “cognitive impairment” and “endometrial mesenchymal stem cells” were recently emerging topics.

**Figure 9:**
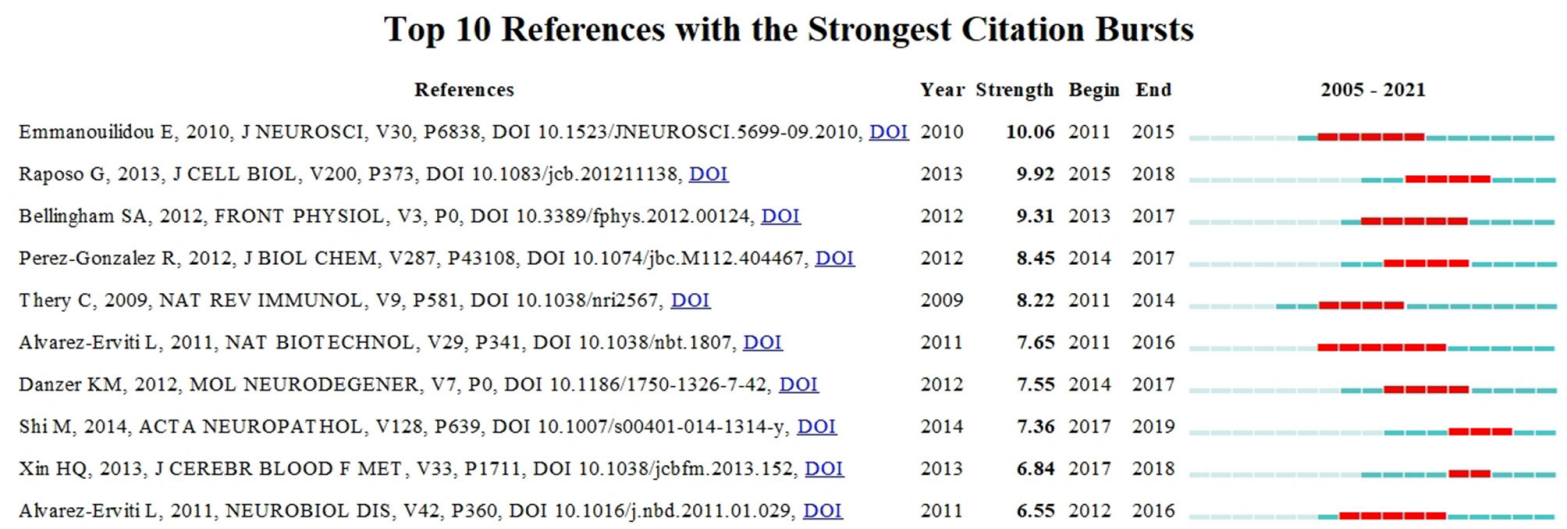
The top 10 references with the strongest citation bursts on exosomes in the field of neuroscience during 2005–2021.

“References with citation bursts” referred to the corresponding articles that had been frequently cited during a period of time in a field. As exhibited in Figure 9, red segments represented “burst” years, and light blue represented the year of publication. Most of the articles had citation bursts from 2014 to 2016. The highest citation burst strength for Emmanouilidou E et al. showed rapid dissemination and significant contribution of their works on exosomes in the field of neuroscience (31).

**Figure 10:**
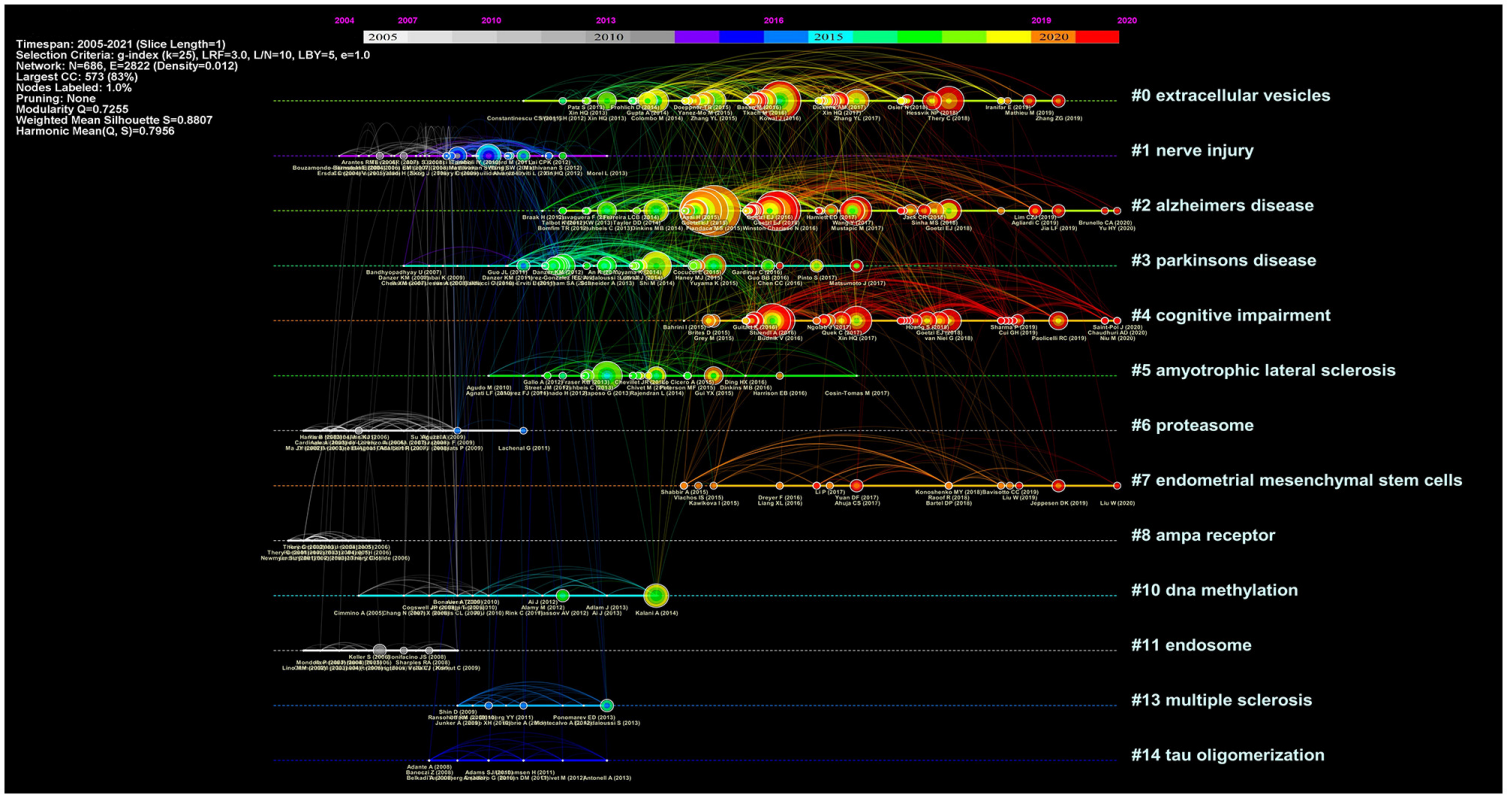
A timeline view of co-citation network about exosomes in the field of neuroscience.

To visually reflect the publication time characteristics of co-cited articles, these important documents in each cluster were placed on the same horizontal timeline (Figure 10). The colors of each outline corresponded to the time slice color at the top. The number of nodes reflected the importance of each cluster, and their sizes were determined by co-citation frequency. Furthermore, the time span of documents in each cluster showed their significance in a related field. To sum up, clusters of Alzheimer's disease and cognitive impairment seemed to be very important and kept on developing. “Nerve injury,” “Parkinson's disease, “proteasome,” and “DNA methylation” had a relatively long time span, which could be influential topics about exosomes in the field of neuroscience.

**Figure 11:**
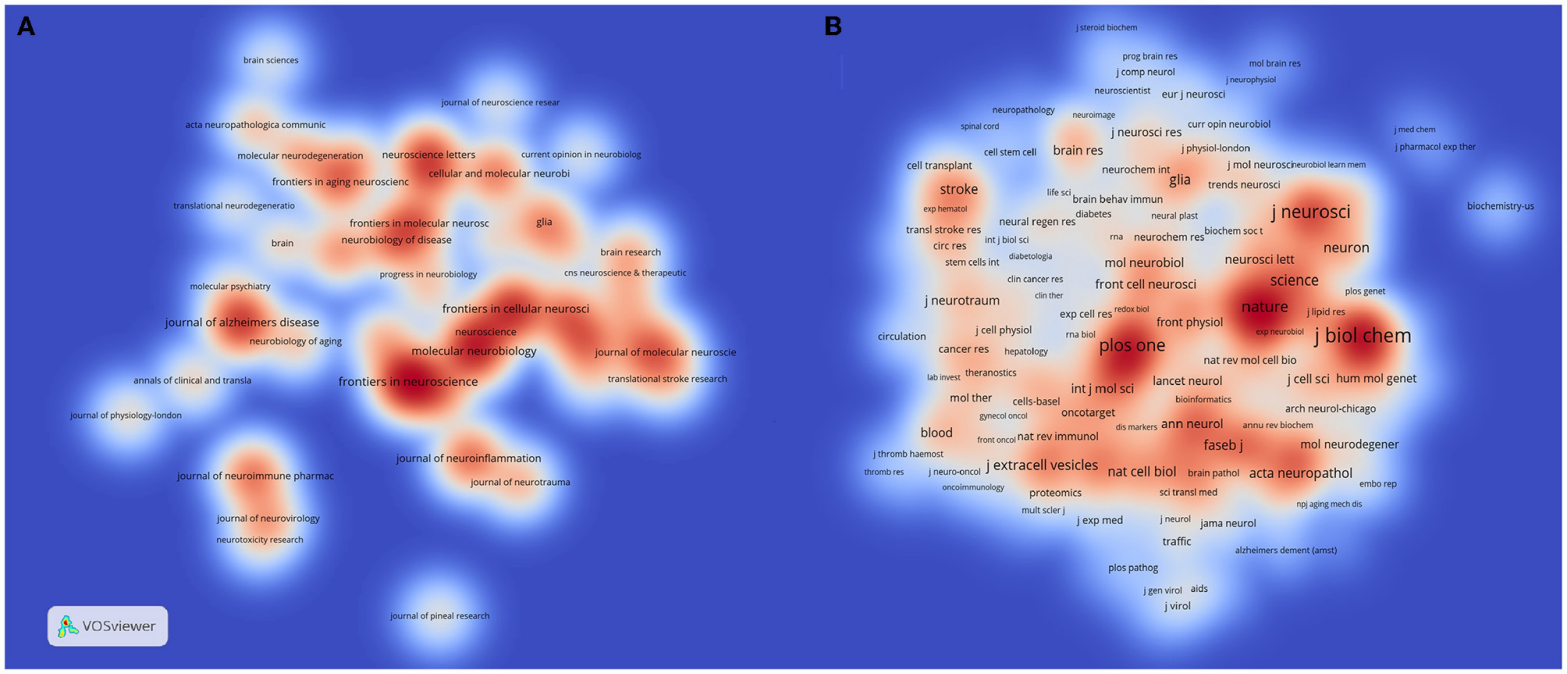
**(A)** Journal density map based on publication number of researches on exosomes in the field of neuroscience. **(B)** Journal density map based on co-citation analysis results on exosomes in the field of neuroscience.

Finally, two journal density maps from VOSviewer were used to show crucial sources on exosomes in the field of neuroscience. Figure 11A displays a density map of citation sources, and in terms of publication number, “Frontiers in Neuroscience,” “Frontiers in Cellular Neuroscience,” and “Molecular Neurobiology” were the top three journals with 48, 34, and 34, respectively. In Figure 11B, the density of a journal was proportional to its contributions in this field. The “Journal of Biological Chemistry,” “Proceedings of the National Academy of Sciences of the United States of America,” “Journal of Neuroscience,” and “PLoS One” provided more than 1,000 co-citations.

## Discussion

Studies on exosomes in the field of neuroscience have made considerable progress within a few years. Although the number of annual publications reached a peak in 2020 and slightly declined in 2021, relevant topics were still popular. The research scopes of these studies were extensive, with many original articles and review studies, and therefore it was necessary to comprehensively analyze and summarize their hotspots and trends. In this regard, we made the first attempt to perform a scientometric study about research on exosomes in the field of neuroscience through statistics and visualization methods.

### Key Word Analysis Based on Titles

From our key word analysis results based on titles, in addition to words related to exosomes, Alzheimer's disease and mesenchymal stem cells were top two words with high frequency (≥50). Increasing evidence on the role of exosomes in pathological processes of Alzheimer's disease has shown their significance as biomarker resources and therapeutic targets in managing Alzheimer's disease (32). In addition, as a self-renewing multipotent stromal cell with stable genomes, mesenchymal stem cell can secrete a great number of exosomes, which has emerged as a promising strategy for various nervous system diseases (33, 34). In these regards, it can be inferred that Alzheimer's disease and mesenchymal stem cells have become hot topics about the exosome-related disease and therapeutic approaches in the field of neuroscience.

### Key Word Analysis Based on Biclustering Analysis Results

After a qualitative biclustering analysis by gCLUTO software, related key words were identified and categorized into a cluster. We found that each cluster contained one or two diseases and other key words were closely related to the disease, which could be speculated that each cluster represented a key topic of exosomes in a particular nervous system disease. For instance, astrocytes played multiple roles in the progress of multiple sclerosis lesions, not only by recruiting lymphocytes and confining inflammation but also by forming the glial scar after inflammation (35). Besides, miRNA in astrocytes-derived exosomes was considered as a potential mediator for neuronal plasticity, and on the other hand, exosomes could transfer to neurons by astrocytes, resulting in neuroinflammation through miRNA dysregulation (36). Moreover, activated microglia were able to facilitate the transmission of α-synuclein which was a central component for the pathogenesis of Parkinson's disease (37). As for cluster 4, terms around extracellular vesicles were key research topics in the diagnosis and treatment of Alzheimer's disease.

Clusters 0, 1, and 4 were strongly linked to each other, because they all included neurodegenerative diseases, such as multiple sclerosis, Parkinson's disease, and Alzheimer's disease. As exosomes can easily cross the blood-brain barrier and spread pathogenic proteins associated with neurodegenerative diseases, the diagnostic and therapeutic potentials of exosomes in neurodegeneration have been largely concerned (38, 39). Conversely, clusters 2 and 3 were relatively “isolated” areas, suggesting less investigations on exosomes in stroke, spinal cord injury, and traumatic brain injury.

### Key Word Analysis Based on the Strategic Diagram

In quadrant I, cluster 4 had significantly higher density and centrality than other clusters, so it consisted of high-frequency and closely related key words, which further supported the prominence of topics about Alzheimer's disease-related exosomes. On the other hand, mature researches about exosomes in Alzheimer's disease also highlighted the necessity and importance of expanding related research directions. Clusters 0 and 1 were considered as widespread issues on exosomes in the field of neuroscience because of their positive centrality in the fourth quadrant. From the prospective of scientometrics, it seemed that multiple sclerosis and Parkinson's disease would be valuable directions for future exosome-related research. Indeed, these neurodegenerative diseases had a long preclinical period with progressively irreversible pathology, and exosomes had been identified as key pathological contributors to them, as well as acting as the diagnostic biomarker for their early detection (40, 41). Topics about exosomes in stroke, spinal cord injury, and traumatic brain injury were in the third quadrant, which might be immature, unpopular, or controversial studies. This outcome might partly be explained by the acute and traumatic characteristics of these diseases, and researchers had mainly focused on the therapeutic role of exosomes (42–44). However, with the development of exosome-based delivery technology, exosome therapy for acute central nervous system injury might make a major breakthrough in the immediate future.

### Co-citation Network and Timeline Visualization Analyses

The co-citation network showed that the top 3 most-cited articles were all related to Alzheimer's disease, and further reflected the tremendous influence of studies about exosomes in Alzheimer's disease, which could be considered as significant fundamental research in this field. From the results of citation bursts, a study by Emmanouilidou E et al. reached the highest citation burst strength and demonstrated that cell-produced α-synuclein could be secreted *via* exosomes in a calcium-dependent manner (31). We found that this theory was largely cited by research about exosomes in neurodegenerative diseases, especially Parkinson's disease. The insights gained from this work might contribute to our understanding of existing knowledge in related fields.

In the clustering analysis results of co-citation network and timeline visualization, a cluster of “cognitive impairment” emerged with growing influence in recent years. To be sure, previous works primarily focused on the role of exosomes in Alzheimer's disease-related cognitive impairment, and over recent years, a few studies had explored the effects of exosomes in some other cognitive impairments, such as mild cognitive impairment (45), cancer-related cognitive impairment (46), and HIV cognitive impairment (47). These observations might support our findings that exosome-related researches have gradually expanded from Alzheimer's disease to some other cognitive impairments.

### Limitations

Nonetheless, we were aware of several latent limitations that might influence the results of this scientometric study. Current work only searched Web of Science Core Collection databases, and some articles could have been missed inevitably. A majority of included studies were original articles and reviews, so these materials might contain some repetitive studies. Included publications were written in English, which might be linguistically biased.

## Conclusions

Alzheimer's disease-related research remained hot in the relevant field till now, and researchers had expanded the scope to more cognitive impairments in recent years. Noticeably, exosomes in the field of neurodegenerative diseases, including multiple sclerosis and Parkinson's disease, displayed promising performance from scientometric analysis results, which might attract more attention in the future. While exosomes in stroke, spinal cord injury, and traumatic brain injury had not been insufficiently investigated, with continuous improvement in exosome-based delivery technology, these subjects might make a breakthrough in terms of therapeutic innovations in the immediate future.

## Data Availability Statement

The original contributions presented in the study are included in the article/Supplementary Material, further inquiries can be directed to the corresponding author.

## Author Contributions

JL and YZ: conception, design, and draft preparation. MP and QG: literature search and collection. XL: data check. JL: software, visualization, and formal analysis. XL, MP, and QG: revision. YZ: supervision and funding acquisition. All the authors listed have approved the manuscript.

## Funding

This research was funded by the National Natural Science Foundation of China [81704135], the Foundation of Henan Educational Committee [18A360012], and the Technological Project of Henan Province [192102310424].

## Conflict of Interest

The authors declare that the research was conducted in the absence of any commercial or financial relationships that could be construed as a potential conflict of interest.

## Publisher's Note

All claims expressed in this article are solely those of the authors and do not necessarily represent those of their affiliated organizations, or those of the publisher, the editors and the reviewers. Any product that may be evaluated in this article, or claim that may be made by its manufacturer, is not guaranteed or endorsed by the publisher.
