# Supplementary material for: Exosomes in the Field of Neuroscience: A Scientometric Study and Visualization Analysis
Source: Front Neurol. 2022 May 17;13:871491. doi: 10.3389/fneur.2022.871491 (PMC9152024; doi:10.3389/fneur.2022.871491)
Supplement: Supplementary file 1 [file Table_1.DOCX]

Supplementary Material

**Supplementary Table 1.** Co-word matrix of high-frequency words of articles on exosomes in the field of neuroscience (partial).

|  | Exosomes | Extracellular vesicles | Biomarkers | Alzheimer′s disease | ... | Central nervous system |
| --- | --- | --- | --- | --- | --- | --- |
| Exosomes | 490 | 93 | 67 | 67 | ... | 10 |
| Extracellular vesicles | 93 | 186 | 32 | 28 | ... | 8 |
| Biomarkers | 67 | 32 | 113 | 20 | ... | 4 |
| Alzheimer′s disease | 67 | 28 | 20 | 111 | ... | 2 |
| ... | ... | ... | ... | ... | ... | ... |
| Central nervous system | 10 | 8 | 4 | 2 | ... | 20 |

**Supplementary Table 2.** Binary matrix of high-frequency words of articles on exosomes in the field of neuroscience (partial).

|  | 001 | 002 | 003 | 004 | ... | 856 |
| --- | --- | --- | --- | --- | --- | --- |
| Exosomes | 1 | 0 | 0 | 1 | ... | 1 |
| Extracellular vesicles | 0 | 1 | 0 | 0 | ... | 0 |
| Biomarkers | 0 | 0 | 0 | 0 | ... | 0 |
| Alzheimer′s disease | 0 | 0 | 0 | 0 | ... | 0 |
| ... | ... | ... | ... | ... | ... | ... |
| Central nervous system | 0 | 0 | 0 | 0 | ... | 0 |

**Supplementary Figure 1.** Co-citation relationship.
