# Supplementary figures and images for: Exosomes in the Field of Neuroscience: A Scientometric Study and Visualization Analysis
Source: Front Neurol. 2022 May 17;13:871491. doi: 10.3389/fneur.2022.871491 (PMC9152024; doi:10.3389/fneur.2022.871491)

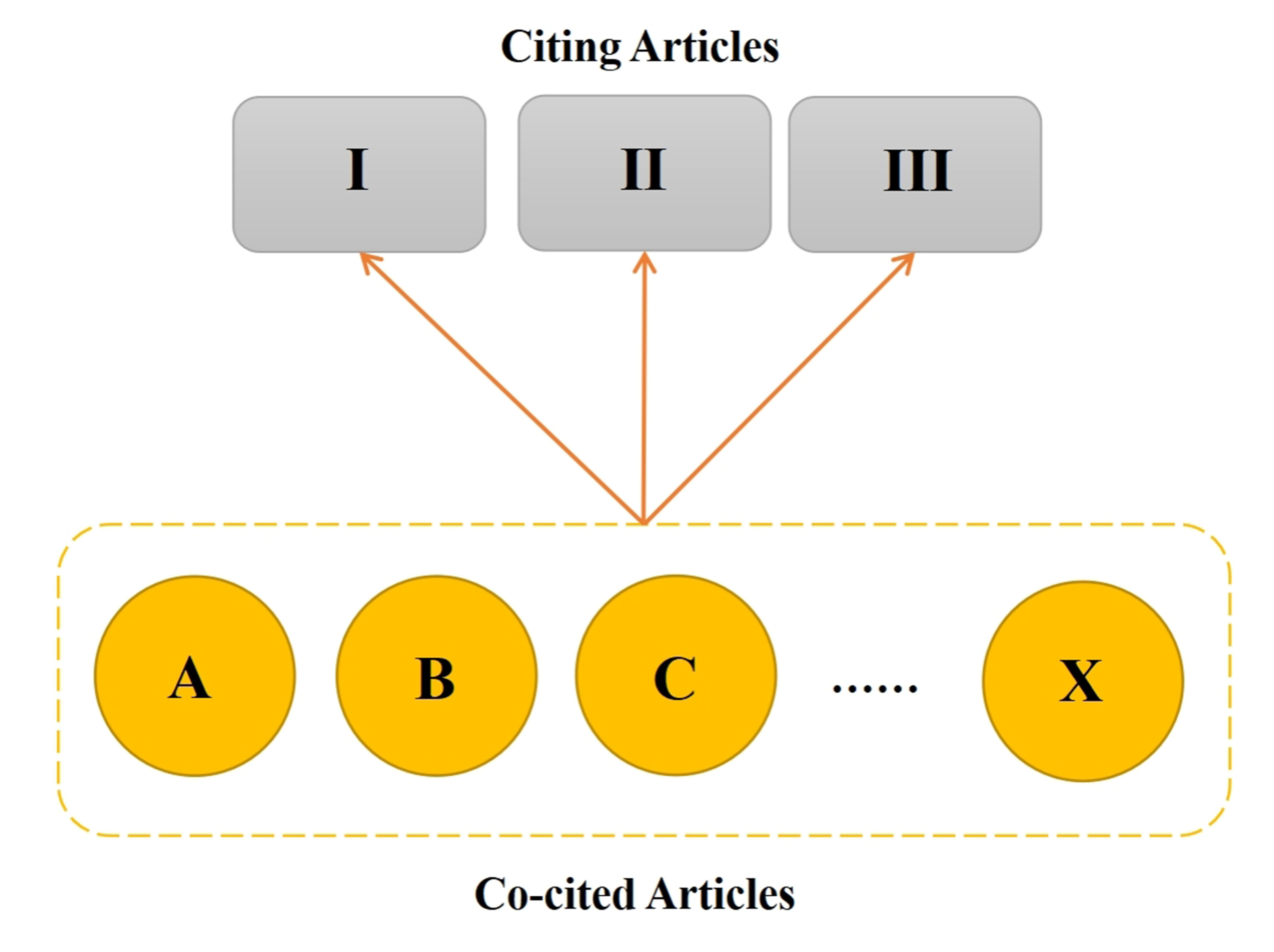

Supplement: Supplementary file 2 [file Image_1.jpg]
